# Surgical proficiency decreases the rate of healing abnormalities using anterior transobturator mesh in cystocele women

**DOI:** 10.12688/f1000research.10012.1

**Published:** 2016-11-10

**Authors:** Jin-Sung Yuk, Yong Jin Kim, Kyong Wook Yi, Jun-Young Hur, Jung-Ho Shin

**Affiliations:** 1 Department of Obstetrics and Gynecology, College of Medicine, Gyeongsang National University, Gyeongsang National University Changwon Hospital, Changwon, Korea, South; 2 Department of Obstetrics and Gynecology, Korea University College of Medicine, Seoul, Korea, South

**Keywords:** Cystocele, Surgical mesh, Treatment outcome

## Abstract

**Aims:** The objective of this study is to report the outcomes of cystocele repair with anterior transobutrator mesh kits.

**Methods:** 119 consecutive women with cystoceles were treated between January 2006 and November 2010 by a single surgeon at a university hospital using the anterior transobturator mesh kit procedure. Postoperative follow-up visits were scheduled at 1, 6, and 12 months after surgery.

**Results:** A total of 114 women who were operated on with the anterior transobturator mesh kit completed 12 months of follow-up. The population had a mean age of 65.8 ± 7.0, a body mass index of 25.1 ± 3.0, and a parity average of 4.0 ± 1.7. An overall anatomic cure was reported for 108 patients (94.7%). The Ba point of the POP-Q exam used for grading cystoceles decreased significantly from 2.5 ± 1.6 cm to -2.8 ± 0.8 cm after 12 months (P < 0.01). One patient (0.9%) presented with bladder perforation, and five patients (4.4%) showed with healing abnormalities. Surgical case volume was negatively correlated with healing abnormalities after adjusting for age, body mass index, operation time, and parity (P = 0.15).

**Conclusion:** The surgeon’s experience decreases the incidence of healing abnormalities using anterior transobturator mesh in cystocele women. The anatomical cure rate of anterior transobturator mesh is quite good.

## Introduction

According to the Women's Health Initiative, cystocele is a common condition affecting 34.4% of women ^1^. Anterior colporrhaphy is the most common traditional surgical treatment for cystoceles. Although the reoperation rate for anterior colporrhaphy is low, it has a high rate of recurrence, varying from 30% to 70% ^2– 6^. The anterior transvaginal mesh kit is an alternative procedure to anterior colporrhaphy that has been used to repair cystoceles with a lower rate of recurrence ^7, 8^. Prospective observational studies indicate that the anterior transvaginal mesh kit has a success rate of 82.3%–95.8% over one to two years ^6, 9^.

In 2008, the U.S. Food and Drug Administration (FDA) warned of several complications associated with the transvaginal placement of surgical mesh used for pelvic organ prolapse ^10^. Healing abnormalities are the complications of most concern ^10^. Healing abnormality is a general term that includes erosion, rejection, infection, and exposure associated with the use of grafts ^11^. Prior studies have reported that 10.4%–10.5% of surgeries involving an anterior transobturator mesh kit result in healing abnormalities ^9, 12^.

The objective of this study was to document our experience with the implantation of anterior transobturator mesh kits, including a report on the healing abnormalities we observed.

## Materials and methods

### Study design

A retrospective chart review was carried out for 119 women who had undergone cystocele repair using the anterior transobturator mesh kit implantation at the Korea University Guro Hospital between January 2006 and November 2010. The inclusion criterion was second stage or greater cystocele based on Pelvic Organ Prolapse Quantification (POP-Q) ^13^. Exclusion criteria were previous cancer of any pelvic organ, systemic glucocorticoid treatment, immunosuppressed disease, and previous pelvic radiation.

### Description of the operations

**Figure 1.**
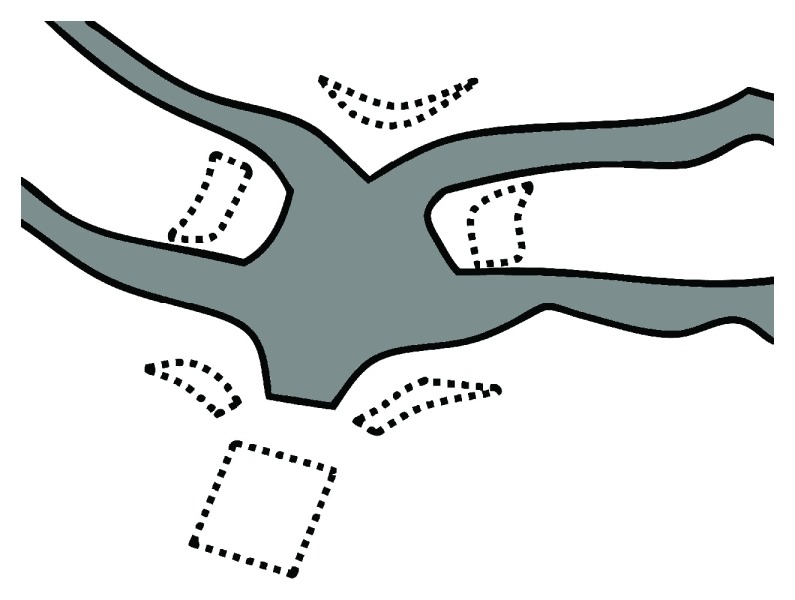
Mesh design. Our unique mesh was designed to fit Korean women and trimmed into a round form. Unbroken line: designed mesh, broken line: derelict mesh.

One of us (J-HS) performed all procedures as the only surgeon and had not performed the intravaginal mesh implantation procedure prior to this study. Preoperative evaluations included taking the patient’s history and performing the POP-Q exam by the surgeon. All patients were implanted with Seratom ^®^ (Serag-Wiessner KG, Naila, Germany) or a Gynecare Prolift ^®^ Anterior Pelvic Floor Repair System kit (Gynecare, Somerville, NJ, USA), as described by Reisenauer *et al.* ^14^. The implantation was performed after at least two weeks of topical estrogen therapy. Our procedure differed slightly from the one described by Reisenauer *et al.* in that 20–30 ml of normal saline was injected without pitressin into the vaginal wall to hydrodissect the pubocervical fascia from the vaginal adventitia. After an incision was created with a scalpel, blunt dissection of the paravesical fossa was conducted using the index finger. Second, the mesh was designed to fit Korean women and was trimmed into a round form ( Figure 1). Third, the mesh was stitched to the pubocervical fascia 1 cm away from the upper and lower margin of mesh, respectively. The Seratom ^®^ and Gynecare Prolift ^®^ Anterior systems kit are similar with only subtle differences in shape. Both are made with type I monofilament polypropylene mesh.

In cases that required hysterectomy or when patients wanted a hysterectomy, a vaginal hysterectomy or total laparoscopic hysterectomy was done. In cases associated with stress urinary incontinence (SUI) symptoms, a urologist was consulted for diagnosis by urodynamic test and treatment. If an operation was needed, the urologist performed a transobturator tape procedure. In cases of second stage or greater rectocele, a posterior Gynecare Prolift ^®^ or posterior repair was conducted. In cases of second stage or greater uterine prolapse, a posterior Gynecare Prolift ^®^ or sacro-spinous ligament fixation was performed.

### Follow-up

Postoperative follow-up visits were scheduled at 1, 6, and 12 months after surgery. The visits included an assessment of side effects and a POP-Q exam. Failure of the procedure was defined as stage two or greater cystocele on the postoperative POP-Q exam. In other words, cure of the cystocele was defined as a stage one or lesser cystocele on the postoperative POP-Q exam. The International Urogynecological Association (IUGA)/International Continence Society (ICS) scale was used to classify healing abnormalities ^15^. Healing abnormalities were defined as category 1–3. The urinary frequency and SUI symptoms were confirmed by patient’s self-reporting.

### Ethics statement

The Korea University Guro Hospital’s Institutional Review Board approved this study. In accordance with Institutional Review Board guidelines, informed consent from the patients was not required because the anonymized data were analyzed retrospectively.

### Statistical analysis

The Statistical Package for the Social Sciences (version 12.0; SPSS Inc., Chicago, IL, USA) was used for all statistical analyses. All statistical tests were two-tailed, and results were considered significant at *P* < 0.05. MetaAnalyst version 3.13 ( http://tuftscaes.org/meta_analyst/) was used for meta-analysis.

In statistical quality control to judge the proficiency of operation, the learning curve-cumulative summation (LC-CUSUM) is a sequential analysis technique used to determine when an operator has reached proficiency. The null hypothesis, H0, is “performance is inadequate.” The alternative hypothesis, H1, is “performance is adequate.” To conduct a LC-CUSUM analysis, four variables are needed: unacceptable failure rate (p1), acceptable failure rate (p0), type I error rate (α), and type II error rate (β). Limit *h* and constant s (always a positive number) were calculated from these four variables. The constant s is subtracted from the cumulative sum during successive performances in the negative direction of y-axis and the value (1-s) is added to the cumulative sum during successive performances in the positive direction of y-axis. LC-CUSUM curves start at zero and are plotted along the x-axis. Two holding barriers that cannot be crossed exist at zero and the limit H1. LC-CUSUM curves must be plotted with only negative y-axis values on a chart. If the LC-CUSUM curve reaches limit *h*, the null hypothesis is rejected. In other words, the trainee attains proficiency. After reaching the limit H1, the LC-CUSUM curve ends and the cumulative summation (CUSUM) curve starts thereafter.

CUSUM is different from LC-CUSUM in several respects. First, CUSUM curves start at zero and are plotted along the x-axis from the x point at which LC-CUSUM ends. Second, the null hypothesis of CUSUM is the opposite of that of LC-CUSUM. In other words, the null hypothesis of CUSUM is “performance is adequate” and the alternative hypothesis of CUSUM is “performance is inadequate.” CUSUM curves must be plotted with only positive y-axis values on a chart. To analyze the cumulative summation curve (CUSUM), the acceptable failure rate (p0) and unacceptable failure rate (p1) were defined as the median value with an upper confidence interval (CI) based on previous studies ^9, 12, 16^. For the LC-CUSUM, the acceptable failure rate (p0) and unacceptable failure rate (p1) were defined arbitrarily as the upper CI + 10% and upper CI, respectively. For these analyses, the type I and type II (α and β) error probabilities were set at 0.05 and 0.20, respectively.

## Results

Pelvic organ prolapse characteristics of patients studied **Variables:** Number, age, f_age (factor age per 5 years), time to cx (time from operation date to complication), time to POP fu (time from operation date to follow up POP-Q exam), time to lastfu (time from operation date to last follow up), op ordinal (order of operation), ht (height), wt(weight), para, BMI, operator, optime (operation time), prolift or seratom, posthtt (previous hysterectomy), vth (co-operation vaginal total hysterectomy), atom (Anterior transobturator mesh), pivs (Posterior Intra-Vaginal Slingplasty), sslf (sacrospinous ligament fixation), tot (Trans-Obturator Tape Sling), Hb (hemoglobin), 2 or 4 atom (Anterior transobturator mesh), 0POPstage (Preoperative Pelvic Organ Prolapse Quantification System Stage), 0ant (cystocele), 0apical (uterine prolapse), 0post (rectocele or enterocele), 0Aa, 0Ba, 0C, 0D, 0Ap, 0Bp, 0TVL (total vaginal length), 0GH (genital hiatus), 0PB (perineal body), 1POPstage (Postoperative Pelvic Organ Prolapse Quantification System Stage), 1ant (cystocele), 1apical (uterine prolapse), 1post (rectocele or enterocele), 1Aa, 1Ba, 1C, 1D, 1Ap, 1Bp, 1TVL (total vaginal length), 1GH (genital hiatus), 1PB (perineal body), recurT (recurrent total prolapse), recurA (recurrent cystocele), recurP (recurrent rectocele), reop (reoperation), urocx (urologic complication), any cx (any complication), erosion, SUI (stress urinary incontinence), frequency, voiding difficulty, itching, constipation, rectocele, dyspareunia, hematoma, rectum injury, anemia, bladder perfo (bladder perforation), other cx (other complication). Click here for additional data file. Copyright: © 2016 Yuk JS et al. 2016 Data associated with the article are available under the terms of the Creative Commons Zero "No rights reserved" data waiver (CC0 1.0 Public domain dedication).

A total of 119 consecutive patients underwent operation for cystocele repair using the anterior transobturator mesh kit. A total of 114 of the 119 patients completed the 12-month follow-up assessment. Seventeen patients (13.0%) did not complete follow-up. Of the 114 patients, a total of 51 patients had undergone Seratom ^®^ (Serag-Wiessner KG) and 63 patients had undergone Prolift ^®^ (Gynecare) repair. The mean ± standard deviation age for the sample of 114 patients was 65.8 ± 7.0 years. The mean body mass index was 25.1 ± 3.0 kg/m ^2^ and the median parity was 4.0 ± 1.7. Nine patients (7.9%) had undergone a hysterectomy prior to this study. No patients had undergone a vaginal mesh operation prior to this study.

Sixty-one patients (53.5%) had greater than second stage apical prolapse, and 21 patients (18.4%) had greater than second stage rectocele ( Table 1). The mean operation time using anterior transobturator mesh kits without a concomitant procedure was 37.3 ± 17.3 minutes. The mean operation time using anterior transobturator mesh kits with a concomitant procedure was 73.9 ± 33.7 minutes. The types of concomitant procedures are reported in Table 2.

**Table 1.** Pelvic organ prolapse characteristics.

Stage	Cystocele	Apical prolapse	Rectocele	Pelvic organ prolapse according to highest stage without distinction of compartment
0	0 (0.0%)	21 (18.4%)	44 (38.6%)	0 (0.0%)
1	0 (0.0%)	32 (28.1%)	49 (43.0%)	0 (0.0%)
2	24 (21.1%)	21 (18.4%)	16 (14.0%)	23 (20.2%)
3	72 (63.2%)	22 (19.3%)	5 (4.4%)	67 (58.8%)
4	18 (15.8%)	18 (15.8%)	0 (0.0%)	24 (21.1%)
Total	114	114	114	114

**Table 2.** Operative characteristics.

Concomitant procedure	Number/total patients (%)
None	15/114 (13.2%)
Posterior transvaginal mesh kit	8/114 (7.0%)
Posterior colporrhaphy	49/114 (43.0%)
TVT-O	13/114 (11.4%)
VTH	39/114 (34.2%)
TLH	5/114 (4.4%)
Sacro-spinous ligament fixation	45/114 (39.5%)

Abbreviation: TVT-O, transobturator tape; VTH, vaginal total hysterectomy; TLH, total laparoscopic hysterectomy.

An overall anatomic cure was observed in 108 patients (94.7%). The Ba point of the POP-Q exam used to grade cystoceles decreased significantly from 2.5 ± 1.6 cm to -2.8 ± 0.8 cm after 12 months ( *P* < 0.01). One patient (0.9%) presented with a postoperative bladder perforation, and five patients (4.4%) presented with healing abnormalities. One patient (0.9%) had an intraoperative hematoma and one patient (0.9%) reported dyspareunia. Sixteen patients (14.0%) presented with urinary frequency, whereas 25 patients (21.9%) presented with SUI. However, all side effects except postoperative bladder perforation and healing abnormality resolved without further treatment. The patient groups who had received Seratom ^®^ and Gynecare Prolift ^®^, respectively, did not differ with regard to the incidence of healing abnormalities. Two patients (1.8%) underwent reoperation.

**Figure 2.**
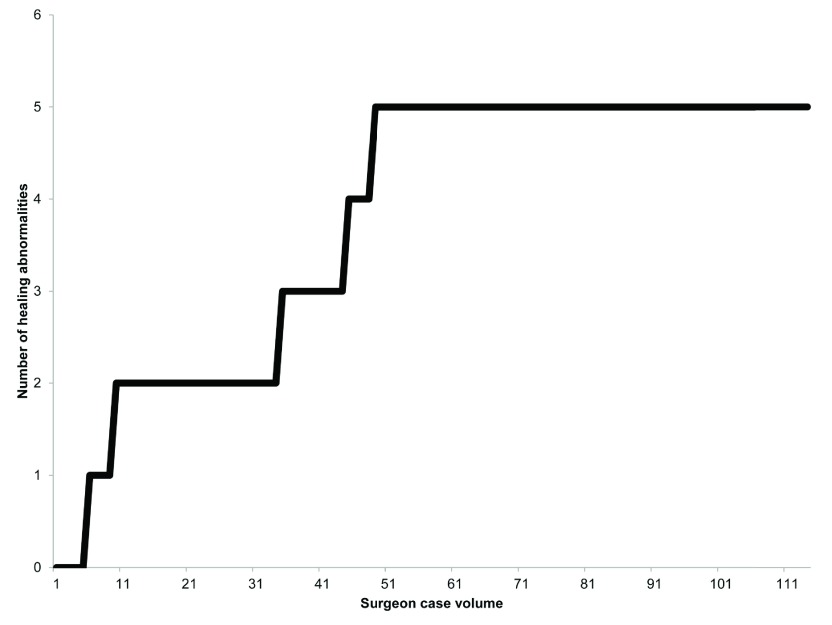
Cumulative sum of healing abnormalities. This figure shows the cumulative sum of healing abnormalities in our cystocele repair using anterior transobturator mesh kits. There did not appear to be any additional healing abnormalities after the fifth healing abnormality.

**Figure 3.**
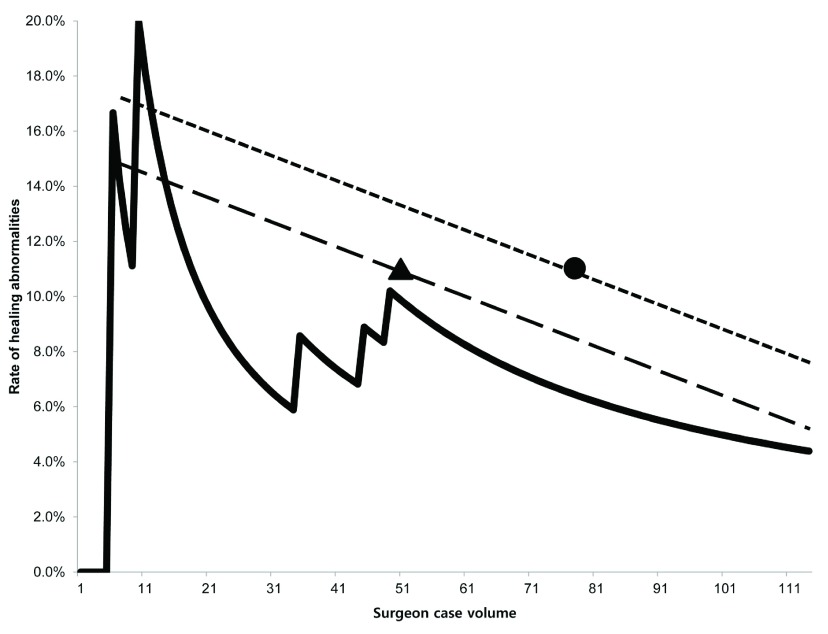
Rate of healing abnormalities per procedure. The graph presents the rate of healing abnormalities per procedure. The continuous healing abnormality rate showed a steady decreasing trend with increasing surgeon case volume. The rates of healing abnormalities in other studies are compared with our result. Triangle: Hinoul *et al.* ^8^, circle: Abdel-Fattah and Ramsay ^11^

Multivariate-adjusted odds ratios (ORs) for healing abnormality are shown in Table 3. The surgeon’s case volume was associated with decreased risk of healing abnormality ( *P* = 0.01). In particular, there did not appear to be any additional healing abnormalities after the fifth healing abnormality (the 49th case volume) ( Figure 2). The continuous healing abnormality rate showed a steady decreasing trend with increasing surgeon case volume ( Figure 3).

**Table 3.** Adjusted ORs for risk of healing abnormalities.

	Healing abnormalities		
	OR *	95% CI	*P*-value
Age (years)	0.93	0.84–1.03	0.15
BMI (kg/m ^2^)	0.79	0.58–1.07	0.12
Surgeon case volume	0.96	0.94–0.99	0.01
Operation time	1.02	1.00–1.04	0.11
Parity	1.10	0.67–1.81	0.70

Abbreviation: OR, odds ratio; BMI, body mass index; CI, confidence interval.

*ORs were adjusted for all variables in the table

Cox & Snell R ^2^: 0.13

**Figure 4.**
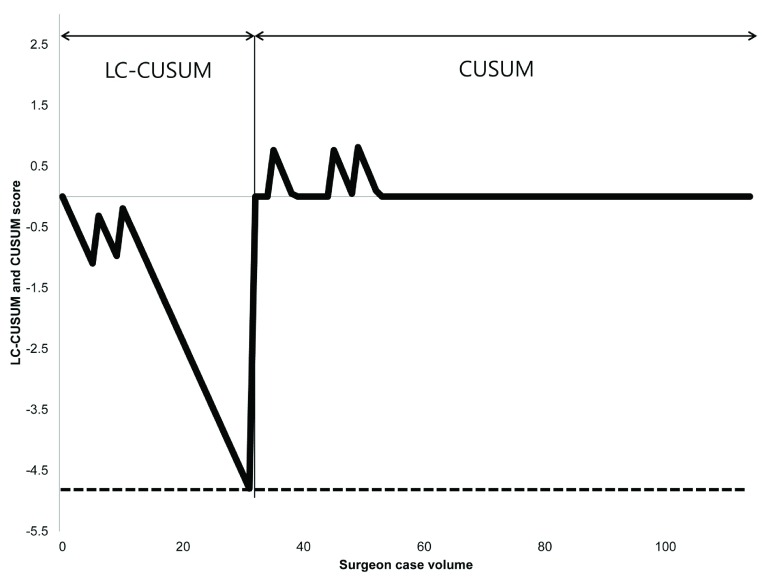
Learning curve cummulative summation curve (LC-CUSUM) and CUSUM curves for healing abnormalities. The acceptable failure rate and unacceptable failure rate of LC-CUSUM are 27.2% (upper confidence interval [CI] + 10%p) and 17.2% (upper CI), respectively, based on a meta-analysis (Hinoul *et al.* ^8^, Abdel-Fattah and Ramsay ^11^). The acceptable failure rate and unacceptable failure rate of CUSUM are 10.5% (median CI) and 17.2% (upper CI), respectively, based on a meta-analysis (Hinoul *et al.* ^8^, Abdel-Fattah and Ramsay ^11^). Dotted line: limit *h* of LC-CUSUM; limit *h* of LC-CUSUM: -4.72; limit *h* of CUSUM: 4.85

LC-CUSUM determine time to gain proficiency and CUSUM monitor quality control after acquisition of proficeincy. LC-CUSUM and CUSUM curves for healing abnormalities are shown in Figure 4. There was an unacceptable failure rate by the 31st procedure based on the LC-CUSUM, after which the curve changed to the CUSUM curve. There was no unacceptable failure rate for healing abnormalities on the CUSUM curve.

## Discussion

In this study, the anatomical cure rate (94.7%) was similar to those of other studies with reported rates ranging from 82.3% to 97.4% ^6, 9, 12^. In other studies, the incidence of healing abnormalities was about 10.4%–10.5% ^9, 12^. In contrast, the incidence of healing abnormalities in our study was much lower at 4.4%. There are several possible explanations for this discrepancy. First, the surgeon’s experience may have affected the incidence of healing abnormalities. In the first 49 cases, the incidence of healing abnormalities was 10.2%. This is comparable to previously reported values (10.4%–10.5%) ^9, 12^. However, the incidence decreased to 4.4% at 114 cases because no healing abnormalities occurred in the later 65 cases. Although several limitations to this study exist, including the fact that a single surgeon carried out the procedures and that different manufacturers' kits were used, Table 3 supports the hypothesis that the surgeon’s experience may have an effect on the incidence of healing abnormalities. If the number of patients in previous studies (respectively, n = 76, n = 48) ^9, 12^ were more similar to the number in this study, there is a possibility that the incidence of healing abnormalities would be lower and more similar to the rate in this study. Additionally, other studies have reported that the surgeon’s experience with prolapse repair reduces the risk of for mesh exposure, including decreased healing abnormalities ^17^. Furthermore, our modified procedure may have decreased the rate of occurrence of healing abnormalities. Sufficient hydrodissection increases the space between the pubocervical fascia and the vaginal adventitia, which makes it possible to preserve the blood supply in the vaginal wall. Pitressin causes vasoconstriction that decreases the blood supply and is used with normal saline in the hydrodissection of anterior colporrhaphy. Our results suggest that using hydrodissection with normal saline but without pitressin might decrease the risk of vasoconstriction.

Because the transobturator mesh kit is designed for use in a Caucasian population, it is generally too large for Koreans. We trimmed the edge of the transobturator mesh kit to anatomically fit the patients and to reduce the risk of mesh folding. To minimize blood supply interference, we chose to use continuous over and over sutures instead of continuous interlocking sutures. Only one other study also successfully decreased mesh exposure with the posterior transobturator mesh kit with a short incision, infra-fascial layer dissection, and no vaginal wall trimming ^18^.

The LC-CUSUM for healing abnormalities indicated that procedural proficiency in terms of healing abnormalities was achieved at the 31st procedure, after which only three healing abnormalities were reported. However, there was no unacceptable failure rate for healing abnormalities on the CUSUM curve. Therefore, quality control was acceptable after proficiency was attained.

Concomitant sacrospinous ligament fixations or posterior transvaginal mesh kits were used in 46.5% of patients, which can cause confounding anatomical outcomes. However, previous studies demonstrated that correction of the posterior compartment with mesh increased cystocele incidence ^19, 20^. Similarly, concomitant sacrospinous ligament fixations or posterior transvaginal mesh kits may increase the incidence of cystocele, or at least not decrease it. Also, 38.6% of patients had concomitant vaginal hysterectomy, which may be confounding. However, one study reported that concurrent vaginal hysterectomy increases mesh erosion ^21^. Therefore, the concomitant operations in this study may have increased the incidences of cystocele or healing abnormality, or at least did not decrease them.

Our study has several limitations. First, we did not investigate the subjective cure rate. Second, our study is based on only a single surgeon’s experience, which limits the generalizability of these results to other surgeons. However, using data from one surgeon reduces confounding factors. Additional reports with a larger sample size are needed.

In conclusion, the surgeon’s experience decreases the incidence of healing abnormalities using anterior transobturator mesh operation in cystocele women. The anatomical cure rate of anterior transobturator mesh operation in cystocele is quite good.

## Data availability

The data referenced by this article are under copyright with the following copyright statement: Copyright: © 2016 Yuk JS et al.

Data associated with the article are available under the terms of the Creative Commons Zero "No rights reserved" data waiver (CC0 1.0 Public domain dedication). 

F1000Research: Dataset 1. **Pelvic organ prolapse characteristics of patients studied**, 10.5256/f1000research.10012.d141554 ^22^.
