# Supplementary material for: Surgical proficiency decreases the rate of healing abnormalities using anterior transobturator mesh in cystocele women
Source: F1000Res. 2016 Nov 10;5:2662. [Version 1] doi: 10.12688/f1000research.10012.1 (PMC5166588; doi:10.12688/f1000research.10012.1)
Supplement: Peer review history

# Peer review: Referee response for version 1

**DOI:** 10.5256/f1000research.10788.r18461

**Published:** 2016-12-14

**Authors:** Sumita Mehta, Anshuja Singla

**Affiliations:** 1 Department of Obstetrics and Gynecology, Babu Jagjivan Ram Memorial (BJRM) Hospital, New Delhi, Delhi, India; 2 University College of Medical Sciences and Guru Teg Bahadur Hospital, Dehli, India

The title is appropriate for the content of the article.

 Abstract is concise and informative.

 The study design and the data have been structured well.

 The objective and conclusion of the study do not match though the title of the study is relevant to the conclusion.

 It is not clear if women with previous failed cystocele repair were included in the present study.

 Page 3, line 3 states: 114 patients of 119 patients completed the 12 month follow up. But the next line states that 17 patients (13%) did not complete the follow up - contradictory statements.

We have read this submission. We believe that we have an appropriate level of expertise to confirm that it is of an acceptable scientific standard.

# Peer review: Referee response for version 1

**DOI:** 10.5256/f1000research.10788.r17533

**Published:** 2016-12-01

**Authors:** Sara Lee

**Affiliations:** 1 Department of Obstetrics and Gynecology, School of Medicine, Ewha Womans University, Seoul, Korea, South

This article is well constructed and analyzed properly. The new information of this article can offer gynecologists good information about the surgery. I recommend this article especially to Asian urogynecologist.

1. The title is appropriate.
2. Abstract - summarized the results well.
3. The authors provide the proper explanantions of the design of this study.
4. The conclusion is based on the study results.
5. They provided the sufficient data.

I have read this submission. I believe that I have an appropriate level of expertise to confirm that it is of an acceptable scientific standard.
